# Flow and Thixotropic Parameters for Rheological Characterization of Hydrogels

**DOI:** 10.3390/molecules21060786

**Published:** 2016-06-16

**Authors:** Mihaela Violeta Ghica, Mircea Hîrjău, Dumitru Lupuleasa, Cristina-Elena Dinu-Pîrvu

**Affiliations:** 1 Department of Physical and Colloidal Chemistry, Faculty of Pharmacy, University of Medicine and Pharmacy “Carol Davila”, Bucharest 020956, Romania; mihaelaghica@yahoo.com (M.V.G.); ecristinaparvu@yahoo.com (C.-E.D.-P.); 2 Department of Pharmaceutical Technology and Biopharmacy, Faculty of Pharmacy, University of Medicine and Pharmacy “Carol Davila”, Bucharest 020956, Romania; office@colegfarm.ro

**Keywords:** hydrogel, rheological models, flow parameters, thixotropic descriptors

## Abstract

The goal of this paper was to design several sodium carboxymethylcellulose hydrogels containing a BCS class II model drug and to evaluate their flow and thixotropic properties. The rheological measurements were performed at two temperatures (23 °C and 37 °C), using a rotational viscometer. The hydrogels were stirred at different time intervals (10 s, 2, 5, 10 and 20 min at 23 °C, and 10 s, 2 and 5 min at 37 °C), with a maximum rotational speed of 60 rpm, and the corresponding forward and backward rheograms were recorded as shear stress *vs.* shear rate. For all hydrogels, the rheological data obtained at both temperatures showed a decrease of viscosity with the increase of the shear rate, highlighting a pseudoplastic behaviour. The flow profiles viscosity *vs.* shear rate were quantified through power law model, meanwhile the flow curves shear stress *vs.* shear rate were assessed by applying the Herschel-Bulkley model. The thixotropic character was evaluated through different descriptors: thixotropic area, thixotropic index, thixotropic constant and destructuration thixotropic coefficient. The gel-forming polymer concentration and the rheological experiments temperature significantly influence the flow and thixotropic parameters values of the designed hydrogels. The rheological characteristics described have an impact on the drug release microenvironment and determine the stasis time at the application site.

## 1. Introduction

Hydrogels are semisolid dosage forms, consisting of three-dimensional networks of water-soluble materials of polymeric, protein, peptidic, colloidal, surfactant, or lipid origin, with a cross-linked structure. They can be formulated in a variety of physical forms, ranging from micro- or nanoparticles to coatings and films applied on solid dosage forms [1,2].

Their applications also cover a vast array, both in clinical practice and experimental medicine. Due to the advanced degree of hydration, their porous structure and low interfacial tension with water or biological fluids, hydrogels have the potential to be used for encapsulation of active pharmaceutical ingredients or therapeutic entities, such as cells [3,4,5], proteins and peptides [6,7,8] and active substances [9,10,11,12,13].

Many of the recently discovered active substances which might be suitable candidates for therapeutic use have a poor bioavailability due to their low water solubility. Therefore, one of the major challenges in developing new medicinal products was to improve the aqueous solubility of these poorly water-soluble drugs, especially of Biopharmaceutical Classification System (BCS) class II active ingredients (high permeability, low solubility) with low molecular weight (MW < 1000 Da) [14,15,16]. By incorporating poorly water-soluble drugs into hydrogels, their aqueous solubility can be enhanced or an extended release can be achieved, thereby increasing the chances of reaching a high drug concentration in a specific organ [17].

Sodium carboxymethylcellulose (NaCMC) is one of the most used polymers in the formulation of hydrogels. Its popularity as a gel-forming polymer is the result of high water absorbent properties, low-immunogenicity and excellent biocompatibility with the skin and mucous membranes. NaCMC primarily maintains an optimal moist environment at lesions level, stimulating extracellular matrix formation and re-epithelialization [18,19,20,21]. It is a derivative of cellulose substituted with carboxymethyl functional groups. Its molecular weight can vary between 90,000 and 2,000,000 g/mol, and has an ether substitution degree varying from 0.6 to 1.0 [22]. NaCMC- based hydrogels are also highly compatible with most drugs. The pH value of the semisolid dosage form can be easily adjusted to ensure the best conditions for the chemical stability of the incorporated drug [23,24].

The therapeutic effects, reabsorption and penetration of the drug are usually improved, compared to hydrophobic ointments. By losing water through evaporation subsequent to their application, NaCMC hydrogels also provide a cooling effect, while maintaining an uniform film on the skin surface [25,26,27].

The study of topical dosage forms flow properties is important from the manufacturers’ standpoint for simple liquids, ointments, creams and pastes. The flow behaviour of semisolids under an applied stress is highly relevant as a quality control tool, helping in maintaining product quality and reducing batch-to-batch variations [28]. Furthermore, it is well known that rheological properties of pharmaceutical systems for topical use influence the release rate of the active pharmaceutical ingredients contained [29,30].

The primary task of mathematical modeling applied in rheology is to reliably predict the rheological properties observed in the laboratories for diluted or concentrated polymeric liquids [31]. By applying mathematical models in the assessment of hydrogel networks and their rheological characteristics, key process and formulation parameters and mechanisms of drug delivery can be identified. Thus, a thorough mathematical understanding of the gel-forming material properties, of the way in which the formulation and process parameters interact, is facilitating the intelligent design of the hydrogel network [32].

The aim of this study was the design of some sodium carboxymethylcellulose hydrogels containing a BCS class II drug model, and the investigation of the rheological behaviour of the resulting experimental hydrogels, in correlation with the formulation variables. The assessment of the flow properties was aimed at the identification of the rheological model that best fit experimental data, as well as at the quantification of the hydrogels thixotropic properties through specific descriptors with biopharmaceutical and technological implications.

## 2. Results

**Figure 1:**
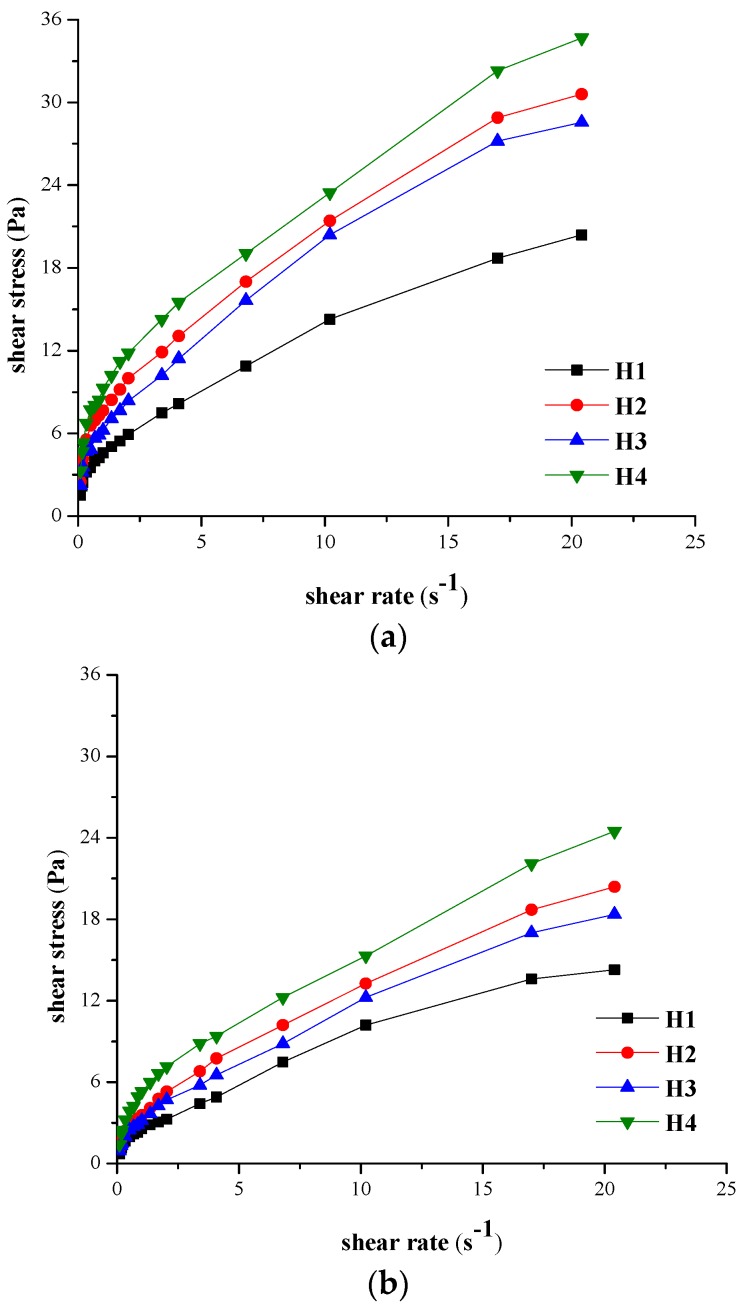
Forward rheograms for hydrogels with minimum concentration of NaCMC analyzed at: (**a**) 23 °C; (**b**) 37 °C.

**Figure 2:**
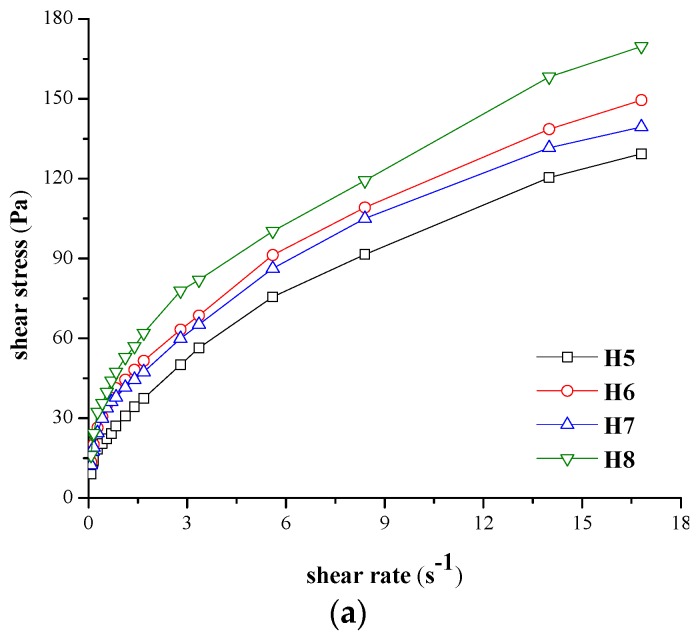

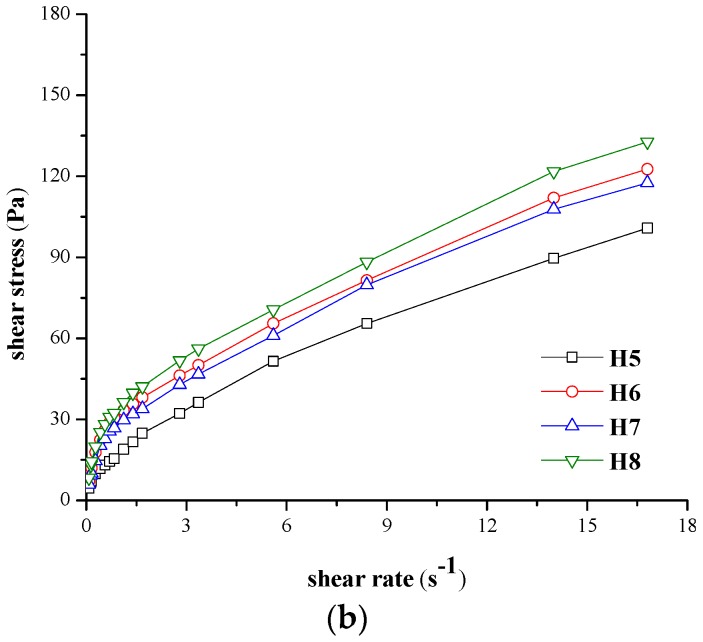
Forward rheograms for hydrogels with maximum concentration of NaCMC analyzed at: (**a**) 23 °C; (**b**) 37 °C.

The forward and backward rheograms were recorded for eight experimental hydrogels, prepared according to the formulas presented in Section “4.2.1. Preparation of the Hydrogels” and coded H1–H8. Figure 1 and Figure 2 illustrate the forward rheograms according to the protocol described in Section “4.2.2. Rheological Measurements”, both at 23 °C and at 37 °C.

The relation between shear stress and shear rate was further analyzed with different rheological models: Ostwald-de Waele (Equation (1)), Herschel-Bulkley (Equation (2)), Bingham (Equation (3)), and Casson (Equation (4)):

(1) $$\tau =K\cdot {\dot{\gamma }}^{n}$$

(2) $$\tau =\tau_{0}+K\cdot {\dot{\gamma }}^{n}$$

(3) $$\tau =\tau_{0}+\eta \cdot \dot{\gamma }$$

(4) $$\tau^{0.5}=\tau_{0}^{0.5}+\eta^{0.5}\cdot {\dot{\gamma }}^{0.5}$$

The significance of the terms from the Equations (1)–(4) is as follows: τ is the shear stress (Pa), $\dot{\gamma }$ is the Shear rate (s^−1^), η is the Plastic viscosity (Pa·s), τ_0_ is the Yield stress (Pa) associated with the critical stress applied for determining the start of hydrogel flow, K is the consistency index (Pa·s^n^) related to the hydrogel viscosity, n is the Flow behaviour index (dimensionless) indicating the non-Newtonian or Newtonian character (n < 1 for a Non-Newtonian pseudoplastic system, n > 1 for a Non-Newtonian dilatant system, and n = 1for a Newtonian system) [33,34,35].

The determination coefficients (”R^2^”) values (Table 1) were used as an indicator to select the hydrogel that best fitted the forward flow profiles.

The values of ”R^2^” specific to Ostwald-de Waele, Herschel-Bulkley, Bingham and Casson models, listed in Table 1, show that the best fit is obtained for Herschel-Bulkley model for all the prepared hydrogels, in this case “R^2^” ranging between 0.9955 and 0.9987 at 23 °C and from 0.9941 to 0.9996 at 37 °C. The descriptors specific to this model are summarized in Table 2 for all systems tested at both temperatures, their significance being previously mentioned.

**Figure 3:**
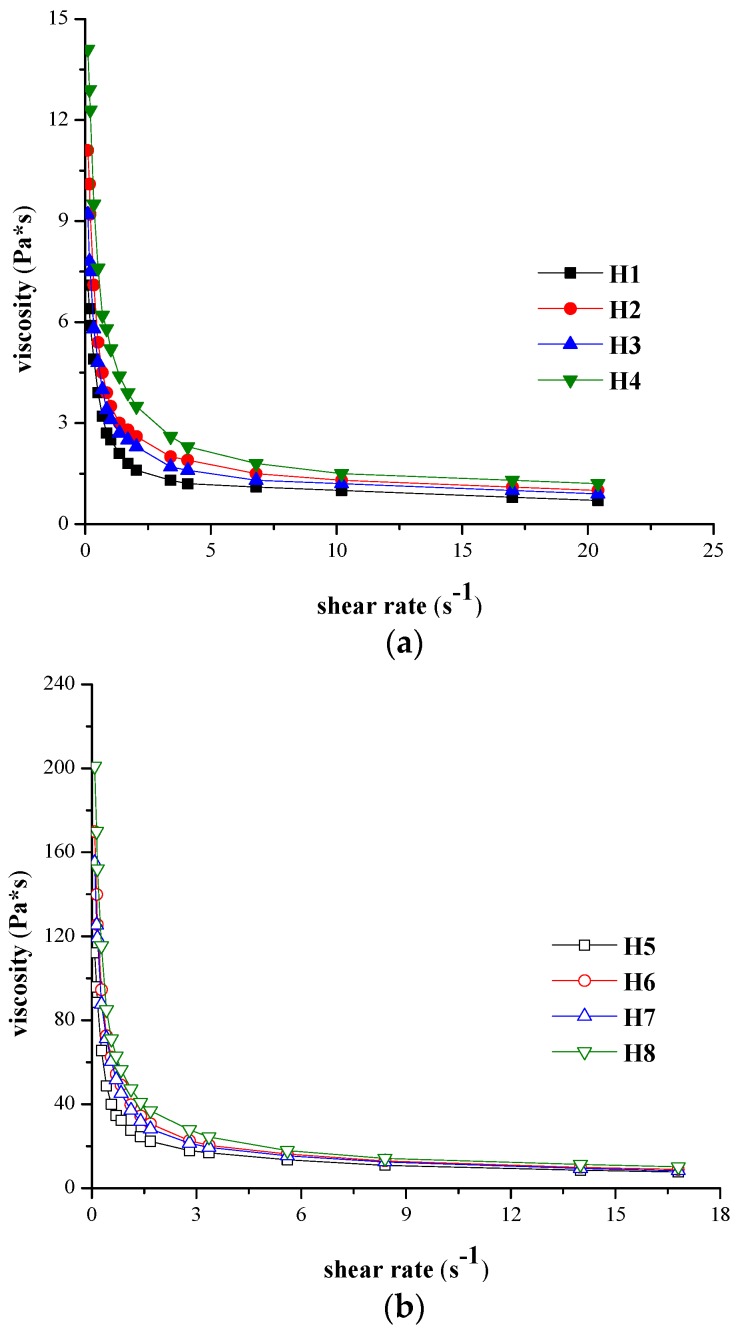
Viscosity *vs.* shear rate for: (**a**) hydrogels with minimum concentration of NaCMC analyzed at 37 °C; (**b**) hydrogels with maximum concentration of NaCMC analyzed at 23 °C.

The hydrogels pseudoplasticity can also be expressed through the forward curves viscosity *vs.* shear rate, presented for exemplification in Figure 3a,b.

In this case, the flow profiles were investigated by fitting the Power law model to the rheological data (Equation (5)):

(5) $$\eta =m\cdot {\dot{\gamma }}^{-n}$$

where “m” and “n” parameters are assessed through the linearization of Equation (5) by double logarithmic method. The “m” parameter is associated with the viscosity obtained for the shear rate of 1 s^−1^ [36].

The power law parameters “m”, “n” and the determination coefficient ”R^2^”, specific to forward and backward rheological measurements (recorded at different stirring times at a maximum rotational speed of 60 rpm) [37] are given for all samples in Table 3 and at both temperatures in Table 4.

Another important issue in the hydrogel rheological characterization is their thixotropic behaviour. The return of the hydrogel to its initial structure is called thixotropy and was assessed by monitoring the viscosity change during the recovery process after shearing [34,37,38,39].

The thixotropic character of the designed hydrogels, determined at both temperatures, was emphasized by the recorded forward and backward rheograms, exemplified for hydrogel H2 analyzed at 23 °C and 37 °C, and hydrogel H8 investigated at 23 °C at different stirring times (10 s, 2 min, 5 min, 10 min, 20 min) for the maximum rotational speed selected for the rheological analysis.

The quantification of thixotropy was accomplished through specific descriptors as thixotropic area, thixotropic constant, thixotropic index and destructuration thixotropic coefficient [34,40,41,42,43,44]:

1. Thixotropy area (hysteresis loop area, S_thix_) is the surface between the forward curve (S_fwd_) and the backward curve (S_bw_) (Equation (6)):
  (6) $$S_{\text{thix}}=S_{\text{fwd}}-S_{\text{bw}}$$
  where S_fwd_ corresponds to a complete hydrogel rheodestruction being correlated to the hydrogel normal manipulation time to expose the drugs incorporated in such formulations to the absorption at the application site, and S_bw_ refers to the recovery of the initial structure by the sheared hydrogel.

The hysteresis area value is an indicator for the degree of system destructuration, higher values for thixotropic area indicating a higher thixotropy.

But backward curve position, in comparison with the forward curve, is depending on the stirring time (t) at the maximum rotational speed that was selected. Thus, Equation (6) can be written as follows:

(7) $$S_{\text{thix}}\left(t\right)=S_{\text{fwd}}-S_{\text{bw}}\left(t\right)$$

Different mathematical relations have been suggested to quantitatively describe the variation of the area included by the backward curve rheogram as a function of the stirring time at the maximum rotational speed selected for the experiment. The equation proposed by Dolz *et al.* has been verified for a series of polymers (Equation (8)):

(8) $$S_{\text{bw}}\left(t\right)=S_{\text{bw}\left(\text{min}\right)}+\left(S_{\text{fwd}}-S_{\text{bw}\left(\text{min}\right)}\right)\cdot e^{-f\left(t\right)}$$

Taking into consideration Equation (8), the relation for determining the thixotropy area becomes:

(9) $$S_{\text{thix}}\left(t\right)=\left(S_{\text{fwd}}-S_{\text{bw}\left(\text{min}\right)}\right)\cdot \left[1-e^{-f\left(t\right)}\right]$$

where S_thix_(t) is the area of the hysteresis loop at a certain stirring moment and S_fwd_ is the area corresponding to the maximum forward or backward curve at the theoretical stirring moment t = 0, S_bw(min)_ is the area under the backward curve at maximum stirring time applied in the experiments, S_bw_(t) is the area under the backward curve at moment “t” of stirring and f(t) is a function depending on the rheological behaviour of the semisolid system studied. This last equation proves the dependency of the thixotropic area on the stirring time at maximum rotational speed. It can be stated that an ideal (minimum) thixotropy area corresponds to a zero stirring time.

2. For different gel-forming polymers used in various concentrations or in various combinations with other components (sodium salt of carboxymethylcelulose, aerosil, sodium carboxymethylcelulose mixed with bentonite), Dolz *et al.* [40,41,42] have found that f(t) becomes:
  (10) $$f\left(t\right)=c\cdot \sqrt{t}$$
  where “c” is the thixotropy constant, a parameter linked to the rate for which the backward area S_bw_(t) reaches its minimum, characterizing the variation in time of the backward area. If the system has a thixotropic behaviour, the value of this constant is higher than 0, as the backward area must decrease with the stirring time increase. Furthermore, only when the thixotropy constant value is finite, the area depends on the stirring time. The more the “c” constant value increases, the faster the system reaches maximum destructuration.
3. The thixotropy index (T_hyst%_) is the relative thixotropy area, expressed as a percentage of the area rheodestroyed by stirring at maximum rotational speed, compared to the backward area (Equation (11)):
  (11) $$T_{\text{hyst}\%}=\left[\frac{\left(S_{\text{fwd}}-S_{\text{bw}}\left(t\right)\right)}{S_{\text{fwd}}}\right]\times 100$$

The higher the value of the thixotropy index, the system becomes more thixotropic. Due to measuring errors in the determination of the shear stress (maximum 5%), during the determination of S_fwd_ and S_bw_(t) areas, only the T_hyst%_ values larger than 5% will be considered. For any result under this value, it can be safely assumed that the gels were non-thixotropic.

4. Another thixotropic parameter, which can be linked to the stirring time at the maximum rotational speed selected is the tixotropic destructuration coefficient (B), determined according to Equation (12):
  (12) $$B=\frac{\tau \left(t_{1}\right)-\tau \left(t_{2}\right)}{\ln\left(\frac{t_{2}}{t_{1}}\right)}$$
  where τ(t_1_) and τ(t_2_) are the shear stress at stirring times t_1_ and t_2_, at maximum rotational speed.

The values for the aforementioned thixotropic descriptors, as well as for the forward and backward areas recorded at different moments during hydrogels stirring at maximum rotational speed and at 23 °C and 37 °C respectively are shown in Table 5 and Table 6.

**Figure 4:**
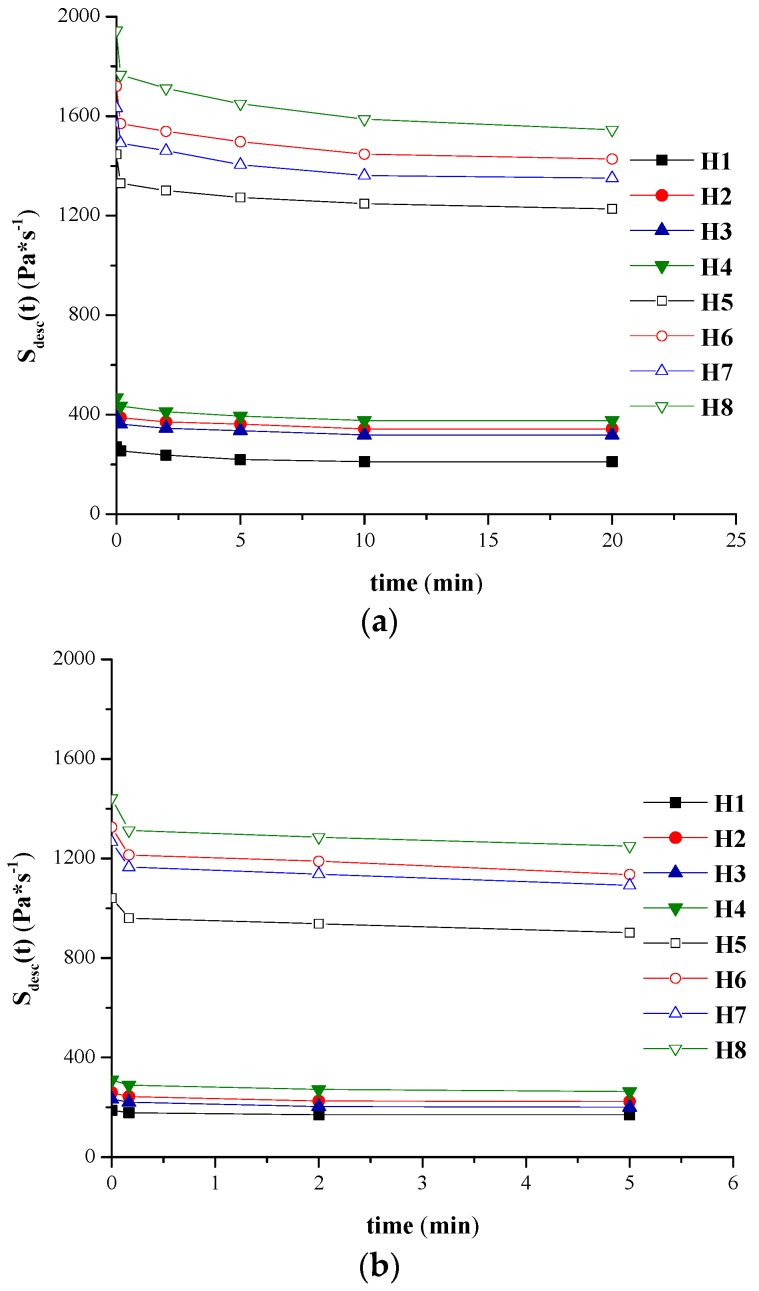
(**a**) S_bw_(t) values *vs.* different stirring times at maximum rotational speed for hydrogels H1-H8 analyzed at: (**a**) 23 °C; (**b**) 37 °C.

**Figure 5:**
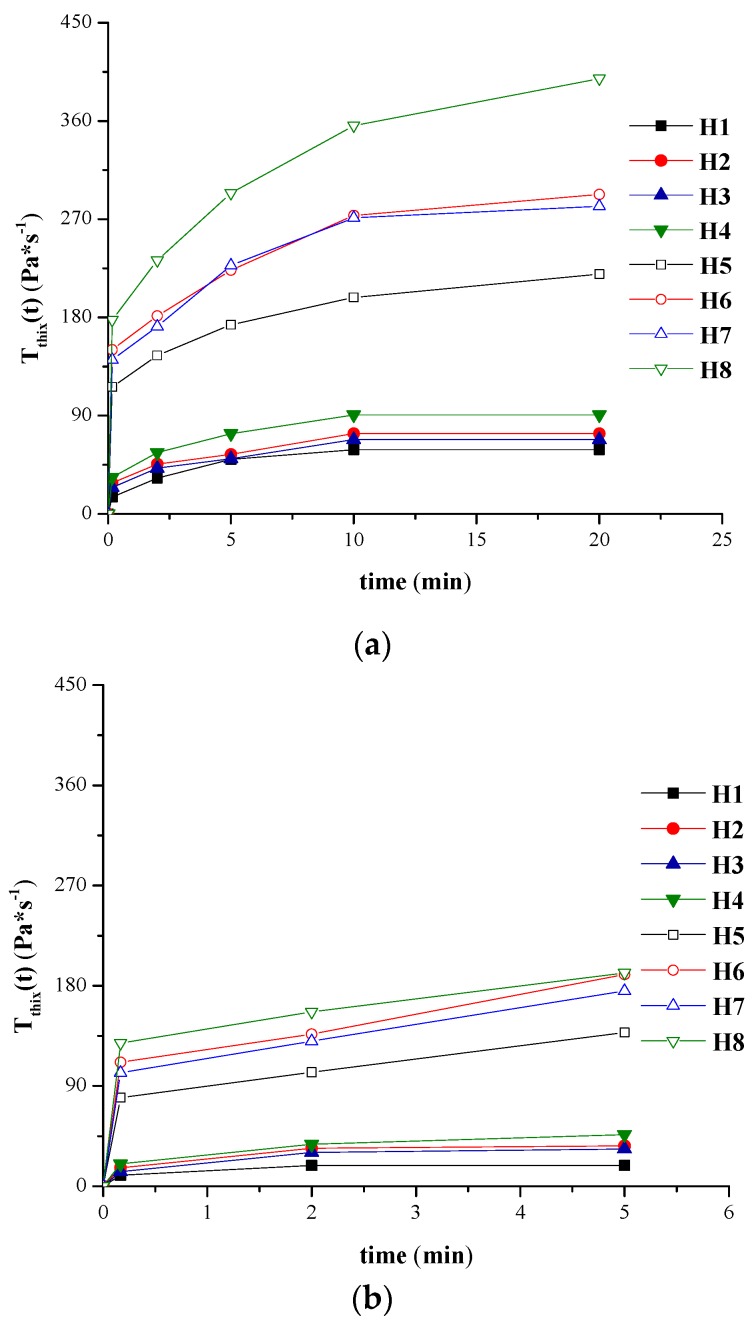
(**a**) Thixotropy area values *vs.* different stirring times at maximum rotational speed for hydrogels H1–H8 analyzed at: (**a**) 23 °C; (**b**) 37 °C.

The graphical conversion of the variation of areas corresponding to the backward curve and of the thixotropy area respectively *vs.* different stirring times at the maximum rotational speed are presented in Figure 4a,b and Figure 5a,b for H1–H8 hydrogels tested at both temperatures.

## 3. Discussion

The influence of formulation factors as well as of the work temperature on different flow parameters and thixotropic descriptors for different NaCMC hydrogels was further discussed. These rheological properties of the prepared hydrogels are strongly influenced by the gelation mechanism. Initially, the water uptake by the NaCMC leads to chain entanglements and solvent entrapment in the spaces formed in the 3D networked structure. As the formation of final NaCMC hydrogels is a consequence of the physical interactions between gel-forming polymer, non-gelling polymer, water and indomethacin as sodium salt, the application of an increased shear stress conducts to an easier partial destruction of the gel network compared to chemically cross-linked hydrogels.

The shapes of the forward rheograms for all the hydrogels, shown in Figure 1 and Figure 2, have similar appearance on the shear rate range applied in the experiments, regardless the concentration of the hydrogels in NaCMC, PEG 400 or PEG 1000. In all cases, the shear stress increased with the shear rate.

The data presented in Table 2 show that the hydrogels exhibit pseudoplastic properties, the value of flow index “n” being less than 1. The flow index values are correlated with the degree of pseudoplasticity, smaller values leading to a marked degree of shear thinning. This behaviour is more pronounced at 23 °C compared to 37 °C, the temperature decrease determining an increase of shear thinning, highlighted by the decrease of ”n” value.

Also, yield stress and consistency index decreased with the temperature increase. Thus, for the hydrogel with minimum concentration of NaCMC, τ_0_ decreases about 2.47 times and K about 1.73 times at 37 °C, meanwhile for the hydrogel with maximum concentration of NaCMC, the above parameters decrease about 1.25 times and 1.58 times respectively. This behaviour is explained by the increased mobility of the polymeric chains and the decrease of polymeric chains clusters life-time.

At a certain temperature, the formulation factor concerning the amount in gel-forming polymer (NaCMC) markedly influences the values of the aforementioned rheological descriptors and determines significant differences corresponding to the two variation levels (1% and 2%). Thus, at the lower temperature, an increase of about 2.42 times for τ_0_ was recorded, respectively of about 3.39–5.84 times at a higher temperature. The consistency index was obviously higher for the hydrogels with a high concentration in NaCMC, at 23 °C the increase being about 7.25–8.80 times, and about 7.65–9.77 times at 37 °C.

As it can be seen from Figure 3, the viscosity decreases with shear stress highlighting also in this way the shear thinning behaviour of the designed hydrogels.

As presented in Table 3 and Table 4, the values recorded for ”R^2^” parameter for the power law model, ranging between 0.9615 and 0.9985, indicate that this rheological model has a good fit with the experimental data obtained at both temperatures and different stirring times at maximum rotational speed.

It can be noticed that for longer stirring times (10 min, 20 min), the “m” parameter remains constant for the hydrogels wth a minimum concentration of NaCMC and various amount of PEG 400 and PEG 1000 (H1–H4), while for the experimental formulations H5–H8, with a double concentration in NaCMC, the polymeric matrix is stronger and has a higher resistance to shear-induced destructuration, which leads to different values of the viscosity for longer stirring duration at maximum rotational speed.

The values of “m” parameter are also strongly influenced by the concentration of the hydrogels in gel-forming polymer and by the temperature at which shearing takes place.

In both representations, shear stress *vs.* shear rate and viscosity *vs.* shear rate respectively, the pseudoplastic properties of all the designed hydrogels is confirmed. The pseudoplastic behaviour with yield stress is a desirable property for the semisolid dosage forms because at high shear rates (e.g., a semisolid product is exposed to when it is taken out of its immediate package, *i.e.*, tube), the gel will flow readily, facilitating the topical administration; in case of low shear rates (when the hydrogel is spread on the site of administration), the material will adopt a higher consistency, recovering its original rheological properties before administration [29,34,36].

**Figure 6:**
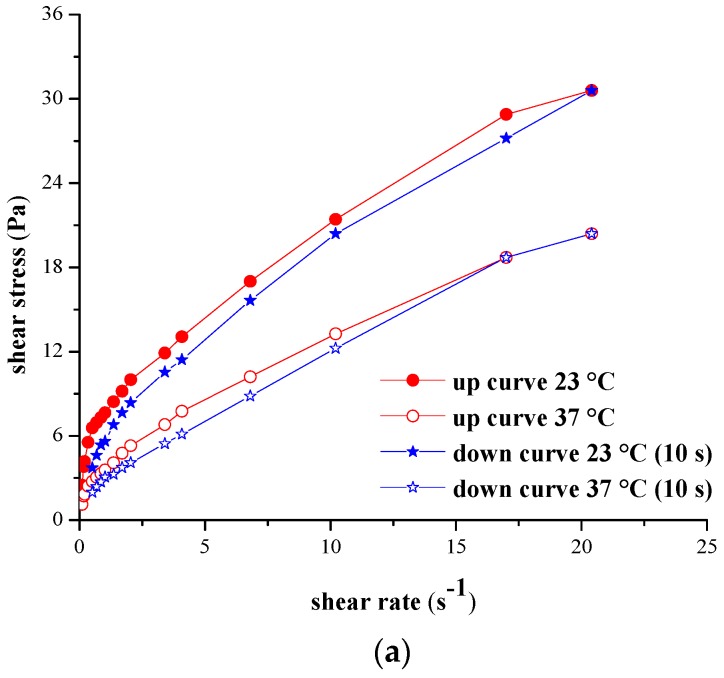

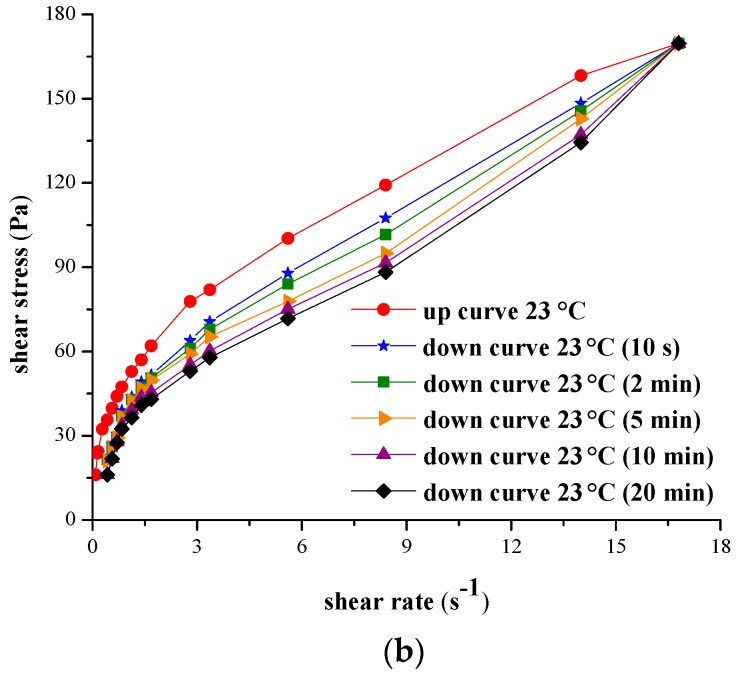
(**a**) Forward and backward (10 s) rheograms for hydrogel H2 tested at 23 °C and 37 °C; (**b**) forward and backward rheograms for hydrogel H8 tested at 23 °C (10 s, 2 min, 5 min, 10 min, 20 min).

The flow patterns recorded in Figure 6a,b indicate that the hydrogels are thixotropic at both temperatures, for the same shear rate, the point on the backward profile corresponding to lower shear stress in comparison with the forward one, obtaining the corresponding hysteresis loops [29].

The shapes of the rheograms illustrated in Figure 6b show that for the maximum rotational speed selected, the increase of the stirring time induces a viscosity decrease, and the backward curve corresponds to decreasing values of the shear stress for the same shear rate.

As expected, for the same stirring period, the values obtained at 37 °C for the areas corresponding to the forward curves, backward curves and thixotropy area are smaller compared to those obtained at 23 °C. Thus, S_fwd_ decreased with 1.43–1.65 times for the formulation with minimum concentration of NaCMC, and 1.28–1.39 times for the hydrogels with maximum concentration of NaCMC. S_bw_ decreased with about 1.27–1.69 times for 10 s, 2 min and 5 min stirring time respectively. Similar to the flow parameters, characteristic both for Herschel-Bulkley and power law model, the formulation factor with a significant impact on the thixotropy characteristics is the concentration in gel-forming polymer. Relevant differences between the values of different area determined for 2% NaCMC hydrogels and those recorded for the 1% experimental formulations were observed. At 23 °C, the thixotropy area for H1–H4 formulations reaches a maximum after 10 min of stirring while at 37 °C maximum destructuration is achieved after 2 min, the thixotropy area remaining constant after this interval; however, for the H5–H8 formulations, a stirring time longer than 20 min (at 23 °C), respectively of 5 min (at 37 °C) is required in order for the systems to destructurate completely.

The values obtained at 23 °C and 37 °C for the thixotropic index (higher than 5%) and those obtained at 23 °C for the thixotropy constant (higher than zero) confirm that hydrogels have a thixotropic behaviour at both working temperatures.

By applying the empiric relationship (Equation (10)) [40,41,42] in the evaluation of the thixotropy constant, values of the “R” correlation coefficient ranging from 0.9598 to 0.9931 were obtained, indicating a good fit of this equation for hydrogels with a complex composition, prepared in this study.

High levels of the thixotropic constant correlated with a fast maximum destructuration were obtained for H5–H7 formulations, for which the NaCMC concentration was the highest. The lower value of “c” obtained for H8 hydrogel, with the same content in gel-forming polymer but maximum concentrations of non-gelling polymers, could be attributed to its considerable higher viscosity (Table 3 and Table 4) compared to H5 hydrogel (“c” max—1.209 min^−½^), requiring a stirring time superior to 20 min for the total destructuration induced by stirring. Comparatively, for H1–H4 formulations, with a minimum concentration of NaCMC, the impact of the stirring period is lower, this being the most obvious for the H1 hydrogel (“c” value is the smallest—0.573 min^−½^).

The thixotropic parameters recorded show that for the hydrogels incorporating a smaller amount of NaCMC, the destructuration takes place for shorter stirring times, compared to the systems including a double amount of gel-forming polymer. This could be explained by the fact that the polymeric matrix is more resistant to the destructuration induced by agitation.

The thixotropic feature is also a quality control parameter, relevant in view of transforming an initially viscous hydrogel into a thin product, easy to spread at the site of administration.

Both the parameters specific to different flow models and the thixotropic descriptors assessed were markedly influenced by the concentration of the gel-forming polymer. The non-gellifying polymers, included in various concentrations in the experimental formulations, influenced the rheological behaviour of hydrogels only in the same batch of systems presenting the same concentration in NaCMC. Their functional role was to modulate the rheological properties of the gel base through their own viscosity, but also through the molecular interactions occuring between these ingredients and the gelling polymer chains.

## 4. Materials and Methods

### 4.1. Materials

Indomethacin (IND) and sodium carboxymethylcellulose (NaCMC) were purchased from Fluka Chemicals Ltd. (Gillingham, UK). Polyethylene glycol 400 (PEG 400), polyethylene glycol 1000 (PEG 1000) and sodium hydroxide (NaOH) were obtained from Merck KGaA (Darmstadt, Germany). Distilled water was used in the preparation of the experimental formulations. All chemical reagents were of analytical grade.

### 4.2. Methods

#### 4.2.1. Preparation of the Hydrogels

The experimental hydrogels were prepared in accordance with the composition presented in Table 7.

The following manufacturing steps were applied for all the formulations: NaCMC was dispersed in an adequate amount of distilled water and left unstirred for two hours to ensure wetting and swelling of the gellyfing polymer. After that, part of the distilled water was gradually added under continuous stirring until an uniform and homogeneous viscous consistency was obtained. PEG 400 was afterwards added under continuous mechanical stirring, followed by PEG 1000 adding, previously dissolved in water. Indomethacin, a non-steroidal anti-inflammatory drug model belonging to BCS class II, was separately dissolved in an appropriate amount of 10% NaOH solution, obtaining the soluble sodium salt, and then it was gently incorporated in the previously prepared hydrogel basis. Using this preparation method, several NaCMC physical hydrogels were obtained, coded as shown in Table 7.

The selection of the non-gelling polymers (polyethylene glycols) in the composition of the experimental hydrogels was based on their good solublility in water and compatibility with NaCMC. Also, polyethylene glycols are excipients widely used in a variety of formulations (topical, parenteral, oral). They are non-irritating and non-toxic in contact with skin. Another rationale for their inclusion in the formulations was the potential to increase the solubility of the poorly water-soluble model drug [45]. Aqueous solutions of PEG adjust the viscosity and consistency of a topical/transdermal formulation. In addition, it is known that PEG 400 is a skin penetration enhancer for indomethacin and other anti-inflammatory drugs [46,47].

Different studies mention NaOH being used as a solubilizing agent for some non-steroidal anti-inflamatory drugs such as piroxicam, for antiviral drugs (acyclovir) or for the antineoplastic agent 5-fluorouracil [48,49,50,51]. The functional role of the 10% sodium hydroxide solution was to solubilize indomethacin by converting it into its sodium salt. In contact with the liquid on the skin surface (with a pH value of 5.5), the drug exists both as sodium salt and acid forms [52]. In these conditions, the thermodynamic activity of the active pharmaceutical ingredient is maximum and allows a maximum absorption rate due to a high concentration gradient.

After preparation at laboratory scale, the experimental hydrogels were diluted with distilled water, resulting in 100 g of hydrogels for each formulation with a final concentration of 1% IND. The appearance of all the experimental hydrogels was homogeneous, clear, yellow in colour, and their pH value was between 7 and 7.5. The hydrogels were equilibrated at room temperature (23 °C) for 24 h to remove the eventual air bubbles incorporated, prior to their characterization by rheological analysis.

#### 4.2.2. Rheological Measurements

The stationary shear flow analysis was conducted with a rotational viscometer Multi-Visc Rheometer (Fungilab, Barcelona, Spain) at 23 °C ± 0.1 °C and 37 °C ± 0.1 °C, and a ThermoHaake P5 Ultrathermostat was attached to the measuring system to keep constant the work temperature [33,34]. After mechanical and thermal equilibration, the hydrogels were sheared at a shear rate specific to TR 9 and TR 10 standard spindles, from 0.1 to 20.4 s^−1^ and from 0.08 to 16.8 s^−1^ respectively, in order to obtain the flow patterns. The shear stress and apparent viscosity were recorded as a function of shear rate. Each hydrogel was stirred for different time intervals (10 s, 2, 5, 10 and 20 min at 23 °C, and 10 s, 2 and 5 min at 37 °C, respectively), at a maximum rotational speed of 60 rpm, and the corresponding forward and backward rheograms were recorded. The rotational speeds varied between 0.3 and 60 rpm for the forward curves, these values being similar to some conditions with biopharmaceutical implications (*i.e.*, the application of the hydrogel at the administration site), and allowed the examination of flow curves with hysteresis loops, as shear rates from the upper ranges are more representative for mixing processes during manufacturing of hydrogels and during administration by spreading the hydrogels on the skin [34]. The rheological parameters were evaluated using Table Curve 2D and Microcal Origin softwares.

## 5. Conclusions

A detailed stationary shear rheometry study was presented, highlighting the influence of formulation factors and work temperature on different flow parameters and thixotropic descriptors for different NaCMC hydrogels. All the designed formulations presented a non-Newtonian behaviour, with shear-thinning and time-dependent properties. The pseudoplasticity and thixotropic character are two properties targeted in the design of semisolid dosage forms, important from biopharmaceutical and tehcnological point of view. The rheology plays an important role in the design, evaluation and optimization of topical/transdermal pharmaceutical products, complementing the complex issue of drug delivery. By correlating the rheological results with the kinetic characteristics, the composition of the gel base can be modulated, designing hydrogels with optimal drug release characteristics and an adequate consistency and stasis time at the administration site.

The rheological analysis applied for the gels with NaCMC, PEG 400, PEG 1000 and Indomethacin, could be generalized to serve as a model for more complex heterogels as well as for other types of topical/trandermal formulations with various aplication sites.

## Figures and Tables

**Table 1:** Determination coefficients (”R^2^”) values specific to different rheological models obtained in relation with forward rheograms for hydrogels tested at 23 °C and 37 °C.

Hydrogel	H1	H2	H3	H4	H5	H6	H7	H8
Temperature 23 °C								
Ostwald-de Waele	0.9939	0.9910	0.9919	0.9899	0.9981	0.9976	0.9969	0.9973
Herschel-Bulkley	0.9978	0.9956	0.9964	0.9955	0.9987	0.9985	0.9976	0.9981
Bingham	0.9667	0.9593	0.9667	0.9577	0.9529	0.9386	0.9345	0.9354
Casson	0.9931	0.9903	0.9920	0.9905	0.9894	0.9827	0.9801	0.9816
Temperature 37 °C								
Ostwald-de Waele	0.9923	0.9968	0.9957	0.9925	0.9990	0.9929	0.9947	0.9937
Herschel-Bulkley	0.9941	0.9991	0.9979	0.9957	0.9996	0.9942	0.9956	0.9953
Bingham	0.9720	0.9760	0.9775	0.9691	0.9740	0.9543	0.9587	0.9544
Casson	0.9898	0.9956	0.9950	0.9827	0.9939	0.9862	0.9879	0.9872

**Table 2:** Herschel-Bulkley parameters obtained in relation with forward rheograms for hydrogels tested at 23 °C and 37 °C.

Hydrogel	H1	H2	H3	H4	H5	H6	H7	H8
Temperature 23 °C								
τ_0_ (Pa)	1.299	2.354	1.935	3.049	3.336	5.889	4.597	6.850
K (Pa·s^n^)	3.045	4.988	4.172	5.729	26.805	36.202	35.131	42.701
n	0.612	0.579	0.624	0.565	0.555	0.490	0.482	0.472
Temperature 37 °C								
τ_0_ (Pa)	0.539	0.881	0.753	1.367	2.115	5.151	3.976	6.114
K (Pa·s^n^)	1.871	2.689	2.321	3.478	15.846	24.494	22.567	26.632
n	0.675	0.660	0.678	0.623	0.648	0.551	0.572	0.548

**Table 3:** Power law model parameters obtained in relation with forward and backward rheograms for hydrogels tested at 23 °C.

Hydrogel	H1	H2	H3	H4	H5	H6	H7	H8
Forward rheogram								
m	4.779	8.221	6.897	9.767	31.340	44.777	42.381	52.903
n	0.531	0.524	0.538	0.559	0.529	0.547	0.534	0.553
R^2^	0.9871	0.9772	0.9852	0.9853	0.9888	0.9941	0.9929	0.9908
Backward rheogram (10 s)								
m	3.138	5.608	4.432	6.583	24.391	34.438	32.881	37.856
n	0.438	0.448	0.374	0.457	0.441	0.521	0.503	0.444
R^2^	0.9718	0.9947	0.9956	0.9971	0.9949	0.9927	0.9871	0.9739
Backward rheogram (2 min)								
m	2.974	5.456	4.281	6.342	24.005	33.902	29.316	36.747
n	0.442	0.450	0.380	0.466	0.446	0.528	0.462	0.449
R^2^	0.9701	0.9943	0.9938	0.9953	0.9946	0.9923	0.9985	0.9777
Backward rheogram (5 min)								
m	2.841	5.325	4.160	6.207	23.548	33.305	27.279	36.137
n	0.455	0.460	0.381	0.471	0.456	0.538	0.457	0.456
R^2^	0.9664	0.9935	0.9908	0.9944	0.9901	0.9917	0.9947	0.9732
Backward rheogram (10 min)								
m	2.739	5.122	4.083	6.124	23.364	32.593	26.667	32.099
n	0.471	0.491	0.406	0.479	0.466	0.549	0.462	0.399
*R*^2^	0.9615	0.9730	0.9912	0.9923	0.9886	0.9902	0.9940	0.9722
Backward rheogram (20 min)								
m	2.739	5.122	4.083	6.124	22.980	32.225	26.415	31.097
n	0.471	0.491	0.406	0.479	0.466	0.552	0.461	0.411
R^2^	0.9615	0.9730	0.9912	0.9923	0.9858	0.9906	0.9932	0.9783

**Table 4:** Power law model parameters obtained in relation with forward and backward rheograms for hydrogels tested at 37 °C.

Hydrogel	H1	H2	H3	H4	H5	H6	H7	H8
Forward rheogram								
m	2.646	3.870	3.330	4.832	18.782	32.683	28.822	36.119
n	0.464	0.492	0.464	0.527	0.448	0.431	0.414	0.459
R^2^	0.9811	0.9841	0.9917	0.9925	0.9917	0.9708	0.9737	0.9780
Backward rheogram (10 s)								
m	2.106	2.910	2.468	3.563	15.576	21.533	20.015	24.273
n	0.424	0.421	0.383	0.406	0.417	0.434	0.419	0.435
R^2^	0.9754	0.9814	0.9845	0.9819	0.9804	0.9941	0.9936	0.9892
Backward rheogram (2 min)								
m	1.994	2.732	2.366	3.412	14.976	20.856	19.525	23.582
n	0.444	0.431	0.401	0.417	0.423	0.434	0.432	0.446
R^2^	0.9639	0.9721	0.9823	0.9761	0.9709	0.9911	0.9888	0.9867
Backward rheogram (5 min)								
m	1.994	2.702	2.201	3.266	14.523	20.330	18.980	22.859
n	0.444	0.432	0.365	0.418	0.437	0.442	0.443	0.440
R^2^	0.9639	0.9672	0.9820	0.9718	0.9649	0.9873	0.9852	0.983

**Table 5:** Thixotropic parameters for hydrogels H1–H8 tested at 23 °C.

Hydrogel	H1	H2	H3	H4	H5	H6	H7	H8
S_fwd_^a,^*	270.299	416.471	386.745	468.170	1446.689	1720.449	1632.662	1943.896
S_bw_^b,^* (10 s)	254.957	388.275	362.508	434.686	1330.407	1570.059	1491.434	1766.209
S_thix_^c,^* (10 s)	15.342	28.196	24.237	33.484	116.282	150.390	141.228	177.687
T_hyst%_^d^ (10 s)	5.676	6.770	6.267	7.152	8.038	8.741	8.650	9.141
S_bw_^e,^* (2 min)	237.573	370.918	344.931	412.038	1301.555	1539.071	1461.101	1711.646
S_thix_^f,^* (2 min)	32.726	45.553	41.814	56.132	145.134	181.378	171.561	232.250
T_hyst%_^g^ (2 min)	12.107	10.937	10.811	11.989	10.032	10.542	10.508	11.947
S_bw_^h,^* (5 min)	220.221	362.004	336.270	394.681	1273.475	1497.124	1404.879	1649.921
S_thix_^i,^* (5 min)	50.078	54.467	50.4758	73.489	173.214	223.325	227.783	293.975
T_hyst%_^j^ (5 min)	18.527	13.078	13.051	15.697	11.973	12.981	13.951	15.123
S_bw_^k,^* (10 min)	211.563	342.839	318.686	377.426	1248.342	1447.025	1361.398	1588.079
S_thix_^l,^* (10 min)	58.736	73.632	68.059	90.744	198.347	273.424	271.264	355.817
T_hyst%_^m^ (10 min)	21.730	17.679	17.598	19.382	13.710	15.892	16.614	18.304
S_bw_^n,^* (20 min)	211.563	342.839	318.686	377.426	1226.898	1427.615	1350.789	1545.026
S_thix_^o,^* (20 min)	58.736	73.632	68.059	90.744	219.791	292.834	281.873	398.870
T_hyst%_^p^ (20 min)	21.730	17.679	17.598	19.382	15.192	17.021	17.264	20.519
C ^r,^**	0.573	0.619	0.599	0.676	1.209	0.819	0.801	0.616
R ^s^	0.9931	0.9828	0.9865	0.9900	0.9643	0.9598	0.9662	0.9714
B ^t^ (2 min)	0.684	0.684	0.684	0.684	1.127	0.563	0.563	1.127
B ^u^ (5 min)	0.999	0.499	0.499	0.999	1.646	1.235	1.646	1.646
B ^v^ (10 min)	0.830	0.830	0.830	1.245	1.709	2.393	2.393	2.735
B ^z^ (20 min)	0.710	0.710	0.710	1.065	1.754	2.339	2.339	2.924

*Symbol key*: a—The area under forward curve; b—The area under backward curve (10 s of stirring at maximum rotational speed of 60 rpm); c—The thixotropy area (10 s); d—The thixotropy index (10 s), e—The area under backward curve (2 min of stirring); f—The thixotropy area (2 min); g—The thixotropy index (2 min), h—The area under backward curve (5 min of stirring); i—Thixotropy area (5 min); j—The thixotropy index (5 min); k—Area under backward curve (10 min of stirring); l—The thixotropy area (10 min); m—The thixotropy index (10 min), n—the area under backward curve (20 min of stirring); o—The thixotropy area (20 min); p—The thixotropy index (20 min); r—The thixotropy constant; s—The correlation coefficient; t, u, v, z—The thixotropic destructuration coefficients computed for a rotational speed of 50 rpm (the first step on the backward curve following the one corresponding to the maximum rotational speed of 60 rpm) after 2 min, 5 min, 10 min and 20 min respectively; *—Measuring unit for forward, backward and thixotropy areas (Pa∙s^−1^); **—Measuring unit for thixotropy constant “c” (min^−1/2^).

**Table 6:** Thixotropic parameters for hydrogels H1–H8 tested at 37 °C.

Hydrogel	H1	H2	H3	H4	H5	H6	H7	H8
S_fwd_^a,^*	188.348	259.634	233.769	309.572	1040.031	1325.405	1267.256	1441.454
S_bw_^b,^* (10 s)	178.495	243.143	220.748	289.547	960.440	1214.052	1165.378	1312.957
S_thix_^c,^* (10 s)	9.853	16.491	13.021	20.025	79.591	111.353	101.878	128.497
T_hyst%_^d^ (10 s)	5.231	6.352	5.570	6.468	7.653	8.401	8.039	8.914
S_bw_^e,^* (2 min)	169.791	225.508	203.421	271.959	937.669	1188.892	1137.027	1284.903
S_thix_^f,^* (2 min)	18.557	34.126	30.348	37.613	102.362	136.513	130.229	156.551
T_hyst%_^g^ (2 min)	9.852	13.144	12.982	12.150	9.842	10.299	10.276	10.860
S_bw_^h,^* (5 min)	169.791	223.365	200.343	263.257	902.030	1135.246	1091.870	1249.791
S_thix_^i,^* (5 min)	18.557	36.269	33.426	46.315	138.001	190.159	175.386	191.663
T_hyst%_^j^ (5 min)	9.852	13.969	14.298	14.961	13.268	14.347	13.839	13.296
B ^t^ (2 min)	0.000	0.684	0.684	0.684	0.563	1.127	1.127	0.563
B ^u^ (5 min)	0.000	0.499	0.499	0.499	1.235	2.058	2.058	1.235

Symbol key: See footnotes of Table 5.

**Table 7:** Composition of the designed hydrogels.

Hydrogel ^1^	H1	H2	H3	H4	H5	H6	H7	H8
IND	1	1	1	1	1	1	1	1
NaCMC	1	1	1	1	2	2	2	2
PEG 400	10	10	20	20	10	10	20	20
PEG 1000	10	20	10	20	10	20	10	20

^1^ The amounts of all components are reported with respect to 100 g hydrogel.

## Abbreviations

The following abbreviations are used in this manuscript:

BCS	Biopharmaceutical Classification System
IND	Indomethacin
NaCMC	Sodium carboxymethylcellulose
PEG	Polyethylene glycol
